# Experiences of family caregivers regarding the health of children with congenital craniofacial anomalies in Colombia

**DOI:** 10.1371/journal.pgph.0006398

**Published:** 2026-09-02

**Authors:** María Mercedes Lafaurie, Lina María Vargas-Escobar, María Clara González, Herney Alonso Rengifo

**Affiliations:** 1 Faculty of Dentistry, Universidad El Bosque, Bogotá, Colombia; 2 Faculty of Nursing, Universidad El Bosque, Bogotá, Colombia; University of Balamand Faculty of Health Sciences, LEBANON

## Abstract

Recognizing the challenges faced by family caregivers regarding the health of children with congenital craniofacial anomalies (CCAs) contributes to strengthening healthcare programs according to patients’ and families’ differential needs. This qualitative study presents the experiences of 25 caregivers of children with CCAs from Bogotá and Cali, Colombia, identified from care registries and consultation statistics provided from public high-complexity healthcare institutions. Grounded in descriptive phenomenology and employing thematic analysis, this research utilized individual interviews and focus group discussions to explore the diagnostic process and its impact, experiences with healthcare services, and the caregiver’s role and daily care activities. Data were analyzed using MAXQDA. qualitative software. Findings highlighted the complexity of caring for children’s health. Challenges included late diagnoses, pessimistic views of the children with CCAs condition by healthcare team members; lack of effective support, information, and guidance from health staff; absence of clear care and referral protocols, and limited access to specific adaptations and timely specialized care for children with CCAs. There were also reduced therapeutic services, and a pronounced gendered caregiving burden when responsibilities fall almost exclusively on mothers. System fragmentation, reflected in deficiencies in communication and a lack of clear, coordinated, and timely pathways of care, as well as the absence of adequate psychosocial support for families, emerged as common structural problems in healthcare services in both geographic settings where this research has been conducted. Gender-sensitive strategies focused on alleviating emotional concerns and the burden of caregiving from diagnosis onward within a patient and family-centered care model are decisive. Improving comprehensive CCAs training for healthcare personnel and adjusting care pathways are suggested to contribute to the implementation of inclusive health programs that address the diverse needs of children and their families.

## Introduction

Congenital anomalies are structural or functional abnormalities that arise during intrauterine life and can be identified prenatally, at birth, or sometimes, only later in childhood. These anomalies can be treated with medical or surgical interventions. Access to such care varies across countries and health system organizations. In childhood, congenital anomalies can result in chronic disabilities that impact children’s lives and those of their families, as well as healthcare systems and society [1].

Globally, congenital anomalies affect 8,000,000 newborns and account for 300,000 deaths annually. Its prevalence in low- and middle-income countries is 64.2 and 55.7 per 1,000 live births, respectively (i.e., 642 and 557 per 10,000 live births, respectively). This variation can be explained by social, racial, ecological, and economic factors [2]. According to a report by the National Institute of Health (INS) of Colombia, the estimated prevalence of congenital malformations was 177 cases per 10,000 live births by 2023, with a rate of 331.4 in Bogotá and 236.2 per 10,000 live births in Cali [3].

Congenital craniofacial anomalies (CCAs) are among the most prevalent pediatric conditions and comprise disorders that affect the function of the head, face, and neck. Prevalence rates and phenotypic characteristics vary widely, and typically involve the skull, the stomatognathic system, the eyes, and/or the ears [4]. There are two major groups that CCAs can be categorized into: those caused by premature closure of the craniofacial sutures—craniosynostosis and faciocraniosynostosis—and those currently considered neurocristopathies, such as first- and second-branchial arch syndromes and orofacial clefts, including cleft lip and palate [5].

In Brazil data were analyzed from the medical records of patients assisted between January 2009 and December 2022 at the Craniofacial Anomalies Rehabilitation Center, resulting 86.9% of cleft lip and/or palate (CL/P), 16.0% of CCAs without CL/P, and 2.8% of syndromes or sequences recognized in the absence of CL/P. CL/P [6]. In Colombia there is no national-level information on CCAs, though there are local reports such as the one by Silva-Giraldo and Porras-Hurtado [7]. Between January 2010 and December 2014, 1.807 patients with CCAs were treated at a private health institution in the central-western region of Colombia. Places such as the mouth and skull represent about 60% of all craniofacial anomalies. The most frequent diagnoses include cleft lip and palate with 323 cases (18.9%), followed by retrognathism (11.7%), ankyloglossia (9.1%) and macrocephaly (9.0%).

A family caregiver of a child with a chronic health issue is responsible for the child’s care, performing or supervising activities of daily living, and seeking to compensate for existing dysfunctions. Care involves understanding and attending to those who are unable to meet all or part of their physical, emotional, and/or affective needs [8]. Recent studies indicate that mothers are the main caregivers of children and adolescents with chronic illness and undertake complex activities outside their usual domestic routines that stem from caring for their children. This can lead to acute and chronic physical and emotional disorders [9].

Caregivers face multiple health needs in caring for children with CCAs. Craniofacial anomalies, especially cleft lip and palate affecting 1 in 500–1 in 2,500 live births worldwide, exert deep influences on virtually every aspect of human development and experience, from fundamental physiological functions such as feeding, breathing, and hearing, to complex psychosocial phenomena including self-perception, social integration, and perception of quality of life. The multifaceted nature of these impacts requires comprehensive, multidisciplinary care approaches across the entire lifespan, involving coordination among diverse medical, dental, allied health, and psychosocial specialists. [10].

CCAs in children can have a significant impact on the psychological well-being of families depending on surrounding factors. Zarit Caregiver Burden Interview (ZBI) was employed to evaluate caregiver burden in 153 family caregivers of children with congenital microtia who underwent auricular reconstruction surgery at a tertiary Xi’an hospital. Among the, interviewees, 26 caregivers (17.0%) reported no burden, 102 caregivers (66.7%) reported mild burden, 22 caregivers (14.4%) reported moderate burden, and 3 caregivers (2.0%) severe burden, Analysis identified different significant factors influencing caregiver burden, including the severity of the lesion, educational level, method of medical payment, household income, communication with others about the child’s condition, caregiver self-blame or guilt, and family support [11].

Various medical stressors were identified in caregivers of children with microsomia. Corresponding suggestions included: providers need to be better informed about CCAs, treatment coordination among specialists, and a preference for a family-cantered approach that emphasizes support, empathy, and clear communication [12].

Feragen et al. [13] described feelings of helplessness and the weight of being responsible for their child’s care in parents of children with rare congenital craniofacial condition developing symptoms that could indicate medical traumatic stress. Implementation of trauma-informed evidence-based resources should be considered in craniofacial care. As Chi Adam et al. [14] reported in Malaysia, caregivers of children with craniofacial diagnoses worried about the financial and work implications of caring for their children and were concerned about their children’s condition being a determinant for them to receive emotional and physical support from healthcare professionals. In a study by Johns et al. [15], caregivers of children with microsomia identified sources of support as online support websites (71%), their spouse/partners (62%), family (57%), and medical providers (48%).

Johns et al. [16] revealed that caring for children with craniofacial microsomia in early childhood can be challenging. All participants reported on the burden of frequent care. It has been described as “just a flurry of a lot of appointments” and therapies. Multiple adaptive responses to the burdens of care, including seeking information about their child’s diagnosis and treatment, primarily through online searches, were reported by all participants. The burdens of care can be reduced by healthcare providers’ understanding of these experiences and advancing in family-centered clinical care. Access to clear, helpful, and trustworthy information can represent a significant source of support. Providing psychological interventions to enhance psychological flexibility (e.g., acceptance and commitment therapy) can benefit caregivers’ and children’s well-being [17].

In 2023, 95.9% of Colombians were affiliated with the General Social Security Health System (SGSSS). Among them, 44.4% belonged to the contributory regime (formal employment sector), while 55.6% were in the state-supported subsidized regime. Beyond daily caregiving, many experiences of caregivers of Colombian children with CCAs are tied to care provided in public health institutions or through Health Benefit Plan Administrators (EAPB) that are state governed [18].

This qualitative phenomenological study explored the experiences of family caregivers regarding the health of children with CCAs in Bogotá and Cali, Colombia, centering its interest on the main challenges faced by them, exploring the impact of diagnosis on the family, their experiences with the healthcare team and the health system, and their role and daily caring activities. Its findings aim to enlighten public healthcare CCAs services and enrich public health policies, providing elements for adequate care pathways and guidelines to improve the quality of healthcare delivery in CCAs services according to patients’ and families’ differential needs. This is the first study in Colombia seeking to understand the healthcare experiences of caregivers of children with CCAs. In this sense, it aims to reduce an important research gap.

## Materials and methods

### Ethics statement

The research adhered to the fundamental principles established in the Declaration of Helsinki, ensuring respect for participants’ rights [19]. Additionally, all participants signed written informed consent for their participation in the study. The research protocol (PCI-2023-015) was approved by the Institutional Ethics Committee of Universidad El Bosque with Official Record # 002-2023, delivered on January 24, 2023.

### Design

This was a qualitative study, using individual interviews and focus group discussions, based on descriptive phenomenology, which helps identify universal themes in patient care [20]. Phenomenology, especially in its descriptive form as articulated by Edmund Husserl, seeks to explore the essence of experiences by setting aside researchers’ preconceptions. Integrating phenomenological insights, public health can develop interventions that are not only evidence-based but also deeply attuned to the realities of the populations attended [21]. Situated on the theoretical proposal of Sundler et al. [22], the descriptive phenomenological approach is oriented towards the identification of human experience, preserving a critical stance and reflecting on the understanding of data and the phenomenon. Implementing thematic analysis, as suggested by the authors, the researchers must adopt an open stance with thoughtfulness to the meaning of the lived experiences currently in focus. When understanding the meaning of lived experiences, we must be aware of the lifeworld and how we interact with others. When researching lived experiences, openness to the lifeworld and the phenomenon focused on must be emphasized.

### Context

Participants were selected from registries of pediatric patients with CCAs provided by public healthcare institutions providing high-complexity care (307 registries from Bogotá and 369 from Cali). Cities of Bogotá and Cali bring together people of different ethnicities (mainly mestizo, Indigenous, and Afro-Colombian individuals). The following section presents a detailed characterization of the participants.

### Participants

A purposive sampling approach was implemented in the study. Consecutive sampling involved systematic enrollment of eligible cases. Data collection was carried out until sample saturation, which occurred when no new elements appeared in consecutive interviews and focus groups. Saturation was considered achieved when interviews no longer yielded new themes or codes. Accordingly, the study aimed to reach a level of saturation that allowed a full understanding of the essence of participants’ reported experiences, without additional information emerging [23]. As decided by authors, in the approach taken in Bogotá, a second focus group discussion was conducted to delve more deeply into the burden of care data.

The inclusion criterion was being family caregivers of children with CCAs attending public entities of the SGSSS in the two selected cities, Bogota and Cali, in the last five years. People with limitations in verbal communication or with sensory alterations that prevented face-to-face communication were excluded. To invite them to participate, participants were contacted by telephone. Eleven of the contacted individuals expressed their decision not to participate because they felt uncomfortable sharing information about their experiences.

Overall, this study included 25 participants: 24 women (22 mothers and 2 grandmothers) and 1 man (father). Thirteen participants were from Bogotá and 12 from Cali. The age between 20–39 is prevalent (16 participants); Technician educational level – post-secondary vocational training offered mainly by public schools during average one year– is the most frequent (17 participants), while four of the participants are professionals and four acceded to primary and secondary education; 12 participants belong to nuclear biparental families (mother, father, and children), 10 to extended families (caregiver, their parents or other family members, and children, with 1 case in which the child’s father is also present), and 3 to single-parent families (mother and children). Of the 25 families, 12 are biparental, while 13 do not have the father present. According to caregivers’ descriptions of their caregiving support resources, the two main sources are their partners (9 cases) and their family networks (9 cases). They also rely on support from their older children. In one case, the caregiver reports limited support. 13 participants are Informal workers, while 5 are housewives and 7 are employees. High levels of labor informality are observed among this group of caregivers, which is related to income instability and, frequently, to family economic hardship, especially when several mothers carry family responsibilities alone.

The ages of their children ranged from 0 to 8 years, with the largest group being between 3 and 5 years. Regarding gender, 16 were girls, and 9 were boys, contrary to what is reported in the literature. In Romania, in craniofacial alterations cleft lip and palate group of children participants in their study, authors reported 46 males versus 39 females, yielding a male-to-female ratio of 1.16:1 [10]. In Brazil, the prevalence of CCAs is higher in boys (53.5%), with 86.9% of the analyzed cases being of cleft lip and/or palate [6]. However, although there is no national measure of CCAs, the situation in Colombia of lip and palate anomaly, from the latest National Oral Health Survey [24], shows higher prevalence in females of cleft lip (0.17% vs 0.03%) and of cleft palate (0.13 vs 0.04%), which could contribute to explaining the atypical gender difference found in this study. This survey included a clinical examination of 20.543 individuals aged 0–79 years from the different regions of the country. Table 1 shows the 10 different anomalies reported by caregivers, with cleft lip and/or palate being the most common (9 cases), plus 1 with Ectrodactyly-ectodermal dysplasia-clefting syndrome which includes a cleft lip and palate condition, and 1 with the Pierre Robin sequence, which is frequently associated with cleft palate. Additionally, another hypothesis that could explain this result can be associated with the possible extra burden that aesthetic implications of CCAs can have for caregivers of girls with the condition, when in Colombian culture women are expected to look beautiful. CCAs can significantly impact facial symmetry and profile balance [25]. Possibly, this situation can be a strong motivation to collaborate in this study, although the topic was not addressed.

**Table 1:** Participants characterization.

Description	Criteria	Number of participants
Participants by city	Bogotá	13
	Cali	12
Participants by gender	Female	24
	Male	1
Participants by age	20-29	12
	30-39	12
	40-49	0
	50-59	|
Participants by educational levels	Primary	3
	Secondary	1
	Technician	17
	Professional	4
Participants by family type	Nuclear family	12
	Extended family	10
	Single-parent family	3
Participants by occupation	Informal workers	14
	Employees	5
	Family housekeepers	6
Participants by relationship with the child	Mother	22
	Father	1
	Grandmother	2
Participants by caregiving support resources	Partner support	9
	Partner + family network support	3
	Family network support	9
	Support from the older children	3
	Limited support	1
Participants by research technique	Individual interviews	10
	Focus group discussions	15
Participants by children’s diagnosis	Cleft lip and/or palate	9
	Craniosynostosis	5
	Microtia	2
	Hydrocephalus	3
	Microcephaly	1
	Ectrodactyly-ectodermal dysplasia-clefting syndrome (EEC syndrome)	1
	Pierre Robin sequence	1
	Saethe Chozen syndrome	1
	Apert syndrome	1
	Muenke’s syndrome	1
Participants by children’s gender	Female	16
	Male	9
Participants by children’s age	0–2	4
	3–5	19
	6–8	2

**Source: the study.**

### Research techniques

The study used individual interviews and focus group discussions. Combining techniques enhances the credibility of the findings and offers a more comprehensive understanding of complex phenomena thought triangulation [26]. The semi-structured interviews aimed to explore the central themes of the phenomenon of interest. This technique enables researchers to uncover thoughts, feelings, and beliefs, and to delve deeply into personal and occasionally sensitive issues [27]. The focus groups permit us to explore aspects for which there is insufficient information and to understand how certain situations affect people [28]. The scripts guiding the interviews and focus groups addressed three general themes: the diagnosis and its impact on the family, experiences with the medical team and the health system, and caregivers’ role and daily caring activities. For each dimension, the script has an open question and the necessary items or ideas to explore during the conversation. For both techniques, a pilot test was carried out to ensure cultural awareness. In S1 Appendix the instruments used are published.

The recruitment period of the study was between 19/09/2024 and 31/03/2025. In both cities, the interviews (average 60 minutes) were carried out privately in participants’ homes, and they could select the meeting point. The face-to-face focus group discussion held in Bogotá (average 70 minutes) took place in a meeting room of the university leading the study. It was decided to conduct the second focus group in Bogotá and the focus group discussion in Cali online to reduce barriers, given that caregivers faced limitations in reconciling household responsibilities with participation in the study. The online focus group discussions (average 90 minutes) were conducted using the Google Meet platform. Face-to-face data collection was conducted by a female nursing professional certified in qualitative research. Online focus group discussion was conducted by two of the authors, both female and professors in qualitative methodology: one with a master’s degree in equality and gender (Bogota focus group discussion), and one with a PhD in nursing (Cali focus group discussion). Not prior relationships existed between researchers and participants. Participants signed written informed consent for their participation in the study.

### Data analysis

In the thematic analysis based on descriptive phenomenology considered in this study, according to Sundler et al.’s proposal [21], interpretation proceeds from the original data to the identification of meanings, themes, and recurrent patterns. This process, as described by authors, is summarized in three steps: 1. Achieve familiarity with the data through open-minded reading; 2. Search for meanings and themes, and 3. Organizing themes into a meaningful wholeness. The analysis aims to understand the complexity of meanings in the data. It involves researchers engaging in data and analysis. The analysis implies a search for patterns of meanings and deciding how such patterns can be ordered into themes [21]. In this study, subthemes act as components of a larger theme to highlight specific dimensions to help understand deeply the realities underlying the narrated experiences.

Data analysis involves a reflective process. Reflectivity in an interdisciplinary research team, as the one in this study (psychology, nursing, and dentistry), allows members to critically examine their own biases, assumptions, and particular styles, encouraging open dialogue and constructive agreements.

The information collected was audio-recorded, transcribed, and subsequently systematized using MAXQDA qualitative analysis software. Three coders participated.

### Methodological rigor

To guarantee the methodological rigor of the study, the following criteria were met: transferability, given the broad data collection across two geographical settings and different diagnoses, as well as providing details about methods and detailed information on the participants and their social and cultural context. Likewise, presenting the accounts which facilitate readers to decide if the results apply on their own situation; credibility, as the analysis was conducted with the support of MAXQDA and included a process of interdisciplinary reflection and agreement from four researchers coming from three different backgrounds (psychology, nursing and dentistry); reliability/dependability, by characterizing the sample and presenting the data collection process, the implemented tools, and the steps of analysis process including a table illustrating categories, themes and subthemes; confirmability, verifying and complementing findings by combining two research techniques, contrasting the findings with other studies, and identifying the scope and limitations of the study [26].

### Strengths and limitations of the study

As for the study’s scope, it addressed 25 cases across two geographical settings and diverse cultural contexts, exploring the experiences of family caregivers caring for the health of children with CCAs, including 10 different anomalies. Combining two research techniques is another strength of the study, which enhances data quality. Although results can support thoughtful transfer to other settings, as Korstjens and Moser [26] point out, they do not support universal generalization, which is a limitation of the study. Another limitation is that the reduced participation of cases from some anomalies doesn´t facilitate a differential interpretation of the experiences from the perspective of the children’s conditions.

## Results

As described above in the Participants section, this study included 25 participants: 24 women (22 mothers, 2 grandmothers) and 1 man (father), 13 from Bogotá and 12 from Cali. Allowing a deeper exploration and understanding of the challenges experienced by caregivers in the context of caring for the health of children with CCAs, the information presented was organized, through a descriptive thematic analysis, into emergent themes and subthemes, corresponding to the three general categories that guided the study: Diagnosis and its impact on the family, Experiences with the healthcare team and the health system and Caregivers’ role and daily caring activities. Table 2 presents Emergent Themes and Subthemes from the General Categories.

**Table 2:** Emergent Themes and Subthemes from the General Categories.

General Categories	Themes	Subthemes
Diagnosis and its impact on the family	Diagnostic challenges	Late diagnosis
		Communication failures
		Pessimistic prognoses
	Emotional consequences	Feelings of guilt and depression
		Lack of counseling and psychological support
		Lack of guidance on basic management of newborns
	Coping and resilience	Spirituality
		Family support
		Information-seeking
Experiences with the healthcare team and the health system	Healthcare system fragmentation	Lack of clear pathways of care for children with CCAs
		Lack of coordination among services
	Referral inefficiencies and organizational barriers	Lack of accessibility and timeliness of specialized care
		Restricted access to therapeutic services
		Limited care during the COVID-19 pandemic
	Positive healthcare support experiences	Positive experiences with medical and rehabilitation services
		Positive Experiences with Healthcare Professionals
		Facilitating role of non-governmental organizations
Caregivers’ role and daily caring activities	Gender overload	Caring as a task for women
		Financial aspects as critical in the experience of caregivers
	Emotional burden and psychological impact off caring	Caring for children with CCAs: an absorbing task
		Physical and mental strain
		Development of children with CCAs as an ongoing challenge

Source: the study.

## 1. Diagnosis and its impact on the family

Recognition of the medical diagnosis of each child’s CCA was deeply mediated by emotions, family dynamics, and communication with healthcare personnel. These are the emergent themes in this domain: 1. Diagnostic challenges, 2. Emotional consequences and 3. Coping and resilience.

### 1.1. Diagnostic challenges

According to reports, in both cities, the diagnostic process was marked by tensions related to the quality, timeliness, and clarity of medical information as well as the lack of guidance, empathy, and support from healthcare personnel, which generated uncertainty and sometimes complicated the coping process. Emerging as subthemes: 1.1.1. Late diagnoses; 1.1.2. Communication failures; and 1.1.3. Pessimistic prognoses.

#### 1.1.1. Late diagnoses.

Experiences surrounding childbirth were worsened by lack of prenatal diagnosis. The perception of late and sometimes ambiguous diagnoses generated distrust toward medical staff and had a strong emotional impact on several families, as one participant noted:

*“I had two ultrasounds, and the doctors said the baby had a big head and was skinny, with low weight. Two months after birth, at the check-up, the paediatrician, who has always seen my older son, was an angel—very calm—and told me, “The baby’s head measures differently. Had anyone identified this?” I said no; I had only noticed a prominent forehead, but I saw no defects in my son—I saw him as perfect. She ordered a test, and that was the first time I encountered the word “craniosynostosis.” I was devastated” (Mother of a child with craniosynostosis, Bogotá).*

#### 1.1.2. Communication failures.

Significant tensions related to the quality, timeliness, and clarity of medical information were evident. Shortcomings in the provision of information emerged as a critical barrier to understanding and accepting the diagnosis in some accounts. The use of technical language made it difficult for caregivers to understand the diagnosis and fostered feelings of mistrust. As one respondent explained, medical information was unclear for her:

*“The diagnosis was explained to me. I was at a loss—I am not a doctor; I am not a nurse; I do not know medicine. He talked about mortality rates—many things I did not understand” (Mother of a child with craniosynostosis, Bogotá).*

#### 1.1.3. Pessimistic prognoses.

In both geographic settings, a recurrent pattern was the non-specialist medical team’s transmission of a disability-centered view of CCAs, with pessimistic prognoses and life-threatening risks, leading families to experience high levels of distress, uncertainty, and guilt. This compromises the emotional stability of caregivers and makes it difficult to build an empowered understanding of the child’s condition, as one of the participants explained:

*“In other words, there were many rejections against the child, against oneself, the father of the family, you know what I mean? In other words, every doctor said, “No, the girl will never walk” (Mother of a girl with microcephaly, Cali).*

### 1.2. Emotional consequences

The birth experiences reveal a significant emotional impact compounded in several cases by the lack of effective communication with healthcare teams that would allow families to express their doubts, experiences, and needs. Emerging subthemes in this domain: 1.2.1. Feelings of guilt and depression; 1.2.2. Lack of counseling, guidance and psychological support; and 1.2.3. Lack of guidance on basic management of newborns.

#### 1.2.1. Feelings of guilt and depression.

Accounts associated with depression and feelings of guilt emerged strongly in almost all cases, adding an additional level of complexity and suffering to the caregiving process. When not addressed by healthcare personnel, these experiences interfere with initial bonding with the child and hinder the acquisition of knowledge about the child’s health condition. This is what a participant related:

*“I thought, ´it was my fault. ´ I thought, ´I didn’t take care of myself, I didn’t take the medicine. ´ Well, I didn’t take the vitamins. ´ It was a clash of worlds, and I thought, ´It was my fault, it was my fault. ´ I felt that I went into a postpartum depression” (Mother of a child with microtia, Bogotá).*

#### 1.2.2. Lack of counseling, guidance and psychological support.

The lack of emotional support during the perinatal period can often negatively affect mothers’ ability to process medical information, build a meaningful understanding of their children’s condition, and develop coping strategies, as described in some cases:

*“I think we should have counseling for each disease—each malformation as well. For example, each clinic should have a help center for certain people, because it is very difficult to be a first-time mother and must experience something so hard” (Mother of a girl with cleft lip and palate, Cali).*

#### 1.2.3. Lack of guidance on basic management of newborns.

Associated with the lack of guidance, some caregivers described situations denoting difficulties in the basic management/care of their newborns, with breastfeeding occupying a central place. This account reveals the situation:

*“There came a time when my son cried, and I did not know whether to hold him or not. I was afraid to breastfeed him; I was afraid something serious would happen, that he would not hear me” (Mother of a child with microtia, Bogotá).*

### 1.3. Coping and resilience

Despite difficulties, the participants employ strategies to cope with the challenges posed by the health condition. The following subthemes emerged: 1.3.1. Spirituality; 1.3.2. Family support; and 1.3.3. Information-seeking.

#### 1.3.1. Spirituality.

In many cases, families’ reactions to the diagnosis also involve the spiritual dimension as a mechanism of resilience and emotional containment, as shown in this testimony:

*“I cried—honestly, at that hospital I cried my eyes out. But in these cases, I say that family unity is something very important, and above all, holding on to God’s hand” (Mother of a child with communicating hydrocephalus, Cali).*

#### 1.3.2. Family support.

Regarding the role of support networks, family spaces were identified in several cases as resources for symbolic and emotional support in Cali and Bogotá, as this participant noted:

*“They have been here for her since she was born—the love of the family. A little girl arrived to bring love and to unite us” (Mother of a girl with microcephaly, Cali).*

#### 1.3.3. Information-seeking.

One resilience strategy considered by several caregivers in dealing with the child’s diagnosis and health condition was the active search for alternative information through various means. These two accounts show us this situation:

*“I kept searching and searching until I found a pediatrician who put me in contact with the specialist” (Mother of a child with craniosynostosis and plagiocephaly, Cali).*

*“So—pardon my words—I did not just accept it blindly. I went all into research and was as persistent as I could” (Mother of a child with microtia, Bogotá).*

## 2. Experiences with the healthcare team and the health system

Care for children with CCAs often involves highly complex services and lengthy intervention processes that require strength and persistence from caregivers, given the barriers that can arise and that hinder effective access to rights. Emergent themes associated with this domain are as follows: 2.1. Healthcare system fragmentation; 2.2. Referral inefficiencies; 2.3. Positive healthcare support experiences.

### 2.1. Healthcare system fragmentation

Although some families were able to establish meaningful links with healthcare entities and reported that, once barriers were overcome, children underwent surgical interventions and treatments that improved their condition, accounts also reported delays, lack of continuity, and poor coordination among key stakeholders, reflecting healthcare system fragmentation. Two subthemes emerged that suggest healthcare system fragmentation: 2.1.1. Lack of clear pathways of care, and 2.1.2. Lack of coordination among services.

#### 2.1.1. Lack of clear pathways of care.

The lack of clear pathways of care was revealed in several testimonies. Lack of experience in managing CCA cases was also mentioned. This not only affects the child’s health but also generates a high level of frustration, emotional exhaustion, and a sense of abandonment among caregivers. This account from a participant illustrates the situation:

*“EAPBs -*providers*- do not have a clear path of how to treat a child with this type of condition. For example, I was sent to three surgeons and, as soon as I went in, the surgeon would say “I don’t treat those cases” and wouldn’t even look at him. And the appointment was over—and I had already waited a month and a half to get it—only to request another surgeon who told me the same thing, and it happened three times. The last surgeon said, “I am training to manage those cases, but the one who really treats them is Dr. [doctor name]. So, I made an appointment with him, and he was the one who performed the surgery. Even if EAPBs lack experience in certain cases, they should at least have a pathway or rely on entities that do treat such cases” (Father of a child with EEC syndrome, Bogotá).*

#### 2.1.2. Lack of coordination among services.

Some of the caregivers identified opportunities to improve support systems, such as the need for strengthened coordination between health services for better access to health care for children, as this quotation reflects.

“*Because if we’re talking about a child with a medical condition, she needs her therapies and her appointments. I mean, you can’t wait six months to take her to see a doctor—even though a general practitioner can obviously issue the referrals for you to authorize. They lack significant coordination.” (Mother of a girl with microcephaly, Cali).*

### 2.2. Referral inefficiencies and organizational barriers

Another critical issue concerns the difficulties associated with accessing necessary and timely specialized care, medical follow-ups, and therapies, which poses an additional challenge to the children’s well-being and the effective management of their health, that were higher during Covid 19 Pandemics. Emerging subthemes in this field: 2.2.1. Lack of accessibility and timeliness of specialized care; 2.2.2. Restricted access to therapeutic services; and 2.2.3. Limited care during the COVID-19 pandemic.

#### 2.2.1. Lack of accessibility and timeliness of specialized care.

Especially in Cali, caregivers describe in greater proportion feeling exhausted when searching for specialized care for their children. Problems with timeliness were observed along with reduced access to medical appointments, as one of the respondents explains:

*Sometimes there is a lot of medical negligence because, for example, my child had to be operated on before she was six months old and the surgery was performed practically at 6 months because they did not authorize it quickly” (Mother of a girl with Muenke’s Syndrome, Cali).*

#### 2.2.2. Restricted access to therapeutic services.

Access to therapies aimed at improving children’s condition was one of the critical issues mentioned in both cities. Several caregivers in both settings reported difficulties obtaining authorization for therapies, which generates a lack of continuity in therapeutic processes. Their children need different kinds of therapeutic services, with speech and language therapy being one of the most important since conditions like cleft lip and /or palate and craniosynostosis largely affect communication abilities. There is no enough compromise of the health system in the rehabilitation of these and other functions as required. Let’s see what a participant noted:

*“My problem today is with therapies: they assign me therapy at an institute, I call the institute, and they say, “We’ll call you in 15 days to schedule the first appointment.” The referral expires, and that is how it has been for a while” (Mother of a girl with cleft lip and palate, Bogotá).*

#### 2.2.3. Limited care during the COVID-19 pandemic.

During the COVID-19 pandemic in both cities, health restrictions led to cancelled appointments, inability to access regular medical check-ups, and implementation of virtual or home-based models that did not always adequately address families’ specific needs, generating concerns about children’s developmental progress. This account from one respondent describes what´s happened in her case:

*“It was complex because there were many restrictions. You would arrive and request an appointment and hear, “No, there’s no availability.” Sometimes the EAPBs would say you cannot enter with a companion or without a mask. These were precautions society needed, of course, but many doors were closed—many programs and many access points to healthcare—so I think it was complex, very complex, on the medical side*” (Mother of a girl with microtia, Bogotá).

### 2.3. Positive healthcare support experiences

Positive healthcare experiences describe ideal scenarios from healthcare models to professionals´ humanized practices. A few services have succeeded in creating clear and organized pathways, achieving a positive impact on children’s health and helping families navigate the care processes. Likewise, the support provided by specialists committed to the well-being of children and their families is highly valued. Also significant is the role of non-governmental organizations, generally geared towards a specific type of alteration, which have stepped in to meet families’ needs where public entities have failed to do so. The next subthemes emerged: 2.3.1. Positive experiences with medical and rehabilitation services; 2.3.2. Positive Experiences with Healthcare Professionals; and 2.3.3. Facilitating role of non-governmental organizations.

#### 2.3.1. Positive experiences with medical and rehabilitation services.

When services are comprehensive, and the multidisciplinary team operates as a network, the benefit to patients and their families is highly valued, as we can see in this account:

*“They have a team of specialists all in one place working together. That’s where the neurologist, the geneticist, and the pediatric specialists are—basically, most of my son’s specialists are there. This works greatly to our advantage. They also have a recreation program, but because of the issue XXX* (Child´s name) *is currently dealing with regarding his ears, we haven’t been able to go swimming—he used to take swimming lessons.” (Mother of a child with microcephalia, Bogotá).*

#### 2.3.2. Positive experiences with healthcare professionals.

Given the multiple hurdles caregivers must overcome to secure care for their children, finding a competent and committed professional is especially valued and sometimes viewed as encountering someone who appears to change or to steer the situation in the right direction. These two participants describe their experiences:

*“That same day he was born, Dr. XXX (name of the doctor) visited him in the ICU and diagnosed him. That same day, his diagnosis was confirmed. Fortunately, Dr. XXX was there; he gave me all the orders for surgery. They performed mandibular distraction, and on his first day of life, he underwent surgery.” (Mother of a child with Pierre Robin Sequence, Cali).*

*“Thank God I arrived at that Hospital; I love Dr. XXX* (name of the doctor). *He has been a light in my life and a guide. I mean, my daughter’s surgery—today you can’t even tell she had a condition (Mother of a girl with cleft lip and/or palate, Bogotá).*

#### 2.3.3. Facilitating role of non-governmental organizations.

The frustration families experience when dealing with inefficient bureaucratic protocols or difficulties accessing specialists translates into emotional strain and a search for alternative solutions—often arranged through external foundations. Especially in Cali, several cases highlighted the importance of non-profit organizations that provided surgical procedures, medications, therapies, or emotional support at no cost, making a significant difference in cases that public entities were unable to solve in a timely manner, as this account reveals:

*“Well, the institution that has welcomed him is XXX (name of the institution). That is the only one I was told of, and I started to inquire. Then they accepted me” (Mother of a child with hydrocephalus, Cali).*

*“The one that helped me the most was XXX Foundation* (name of the institution)*. —for now, the best. Thank God, the therapies have helped her a lot” (Mother of a girl with microcephaly, Cali).*

## 3. Caregivers’ role and daily caring activities

The stories from Bogotá and Cali show that the daily care of children with CCAs is a complex and demanding experience that impacts caregivers’ emotional, social, and work life. In both contexts, the role of the caregiver is constructed as an integral function that ranges from managing medical appointments, therapies and treatments, to providing permanent support in the basic activities of daily living and developing children’s abilities. From this category emerged the next themes: 3.1. Gender overload; 3.2. Emotional burden and psychological impact of caring.

### 3.1. Gender overload

Many of the women in this study, around half as seen in participants´ characterization— have no support from the child´s father in daily life. Gender overload is commonly tied to double burden, unequal distribution of caregiving, financial disparities, and cultural values in the construction of gender roles. Emerging from this theme are these two subthemes: 3.4.1. Caring as a task for women; and 3.4.2. Financial aspects as critical in the experience of caregivers.

#### 3.1.1. Caring as a task for women.

The differences in involvement between maternal and paternal figures reinforce gender patterns that continue to position caregiving as a predominantly female responsibility, and women continue to perform most of the unpaid care and domestic labor. Although fathers’ support in children’s caregiving is reported as important in some accounts, their role is usually more limited or sporadic, as these accounts expose:

*“In our child’s process, I’m the one who stays here. The father comes and goes—he sees him—but he is never truly present” (Mother of a child with hydrocephalus, Cali).*

*“…my husband gets home early, so I tell him: ‘You watch the boy,’ and I get my hair done or get a manicure—an hour or two, maybe once a week—that I take for myself” (Mother of a child with microtia, Bogotá)*

#### 3.1.2. Financial aspects as critical in the experience of caregivers.

A large group of caregivers prefers home working and independent activities to get income, aiming to facilitate their caregiving activities. Economic conditions represent a critical point of convergence in several cases. In both cities, structural challenges reflecting social inequities were found, such as a lack of financial support from the state when required (for example, transport assistance) and limited experience regarding access to social support for the care of their children with CCAs, as some women need. Informal strategies are used for them to solve economic needs. Although there have been effective advances in women’s rights in Colombia, and care has been built into public policy recently, the operative pathways are not yet sufficiently available, as these two accounts reveal:

*“The financial situation is tough—very tough. I tried to apply for a transport subsidy, but I never got an answer—not even when I went to ask in person; I just never heard back. So, you take your child and walk to your appointments when they’re close by, or you find something to sell, something to do—whatever you can manage” (Mother of a boy with craniosynostosis, Bogotá).*

“*No, no, I haven’t had any social support. No, we didn’t have support. I must organize raffles, make puff pastries and rice pudding—anything—to cover his needs” (Mother of a child with communicating hydrocephalus, Cali).*

### 3.2. Emotional burden and psychological impact of caring

The findings reveal that the day-to-day care of children with complex chronic conditions is neither a purely technical nor a mental task, but rather a holistic process that is emotionally charged and socially conditioned. Although children’s health is always a challenge, over time, the focus shifts from medical needs to the growing ability to function more autonomously, including supporting them in school. These are the subthemes emerging from this theme: 3.2.1. Caring for children with CCAs: an absorbing task; 3. 2. 2.Physical and mental strain; and 3.2.3. Development of children with CCAs: an ongoing challenge.

#### 3.2.1. Caring for children with CCAs: an absorbing task.

The testimonies often emphasize the care work linked to maintaining medical and functional routines of children, and they highlight the absorbing and all-encompassing nature of the role, which frequently reconfigures caregivers’ personal and professional projects, as these participants note:

*“You feel frustrated about having studied but not being able to pursue a career—or “fulfill yourself” professionally, so to speak, but as my husband says, the comforting thing is seeing him succeed, even if it’s just in some small way.” (Mother of a child with cleft lip and palate, Bogotá).*

#### 3.2.2. Physical and mental strain.

The emotional pressure placed on the family caregiver in contexts of individualized care is highlighted overall in Bogotá. Living in a big city with more difficulties in getting family support may explain this situation. The subjective impact of caregiving becomes more visible, underscoring the emotional burden of this role and the caregiver’s physical and mental strain, as these accounts reveal:

*“You must keep ten eyes on him all the time, because he talks to you, he gets excited, he laughs, he runs all the time, so you’re constantly saying, “Careful there,” “Watch the edge of the wall,” “Be careful—don’t grab the ball.” There are moments—I won’t deny it—of frustration. There are moments when I say, “I can’t take it anymore.” There are moments when I say, “This is so hard; I can’t handle all this”* (Mother of a child with craniosynostosis, Bogotá).

*“And well, as for the caregiving burden, it was intense—I mean, on top of everything else, I had my son with a disability to look after, including all his medical needs. Back then—my child walks now, but at the time he didn’t—I was constantly carrying him. I have scoliosis, so my back was really taking a beating because I was constantly carrying him—if I wanted to move him from one place to another, I had to lift him up. The caregiving situation involved an overwhelming level of exhaustion—it was brutal.”* (Mother of a child with microcephaly, Bogotá).

#### 3.2.3. Development of children with CCAs: an ongoing challenge.

Caring for a child with CCA is a challenge for caregivers, a daily struggle that is rewarded by seeing their children progress. Concerns are related to their children´s character and personality development, especially when children with CCAs frequently face discrimination that affects their emotional lives, as this account reveals:

*“What is my dream? To be able to fix the situation, to help him grow into a well-adjusted person. He is a normal kid, but nowadays society—or rather, the adolescent world—views his situation differently. It’s a process that feels very external to him because bullying is terrible; bullying and harassment are truly awful. I hope that God gives me the chance to ensure he is doing well—so that when he’s at school or enters high school, he can say, “Yes, I’m okay.” So, I am coming to terms with this process, and I am raising my son to be very strong in that regard (Mother of a child with microtia, Bogota).*

## Discussion

According to the findings of this study, caregiving for a child with CCAs represents for family caregivers a complex and continuous experience, tied to the demands of their child’s condition, from the moment of the diagnosis. Permanently, especially in the child´s early years of life, caregivers, who are their mother in 22 of 25 cases, must interact with a health system that shows structural failings in providing conditions to guarantee the right to health for children with CCAs, considering their specific needs. Economic, social, and gender factors add a greater burden to their experiences, while around half of them are heads of household and high levels of labor informality are observed among this group. Social and psychological challenges emerged in their accounts. The literature reports on various emotional consequences that parents may experience upon the birth of a child with CCAs, which can especially affect the mothers´ psychological state (15–16, 29–30).

Realizing the child´s CCA condition generates a great impact on the caregivers´ lives, loaded with feelings of anxiety, uncertainty, stress, guilt, and depressive symptoms, which emerged especially in the initial stages of diagnosis, which were not always established early on. In addition, participants describe pessimistic prognoses and communication failures from medical professionals in the first stage of their experiences that affected their ability to understand the diagnosis and to cope with the situation. The emotional and psychological impact of the diagnosis is highlighted in previous studies. As Stock et al. [29] expose, the birth of a child with medical and/or developmental needs can carry different psychological and social challenges for caregivers and families. In their study with caregivers of children with microsomia, in response to seeing their baby for the first time, participants reported feelings of joy and love often simultaneously with shock, anxiety, anger, shame, guilt, and worry for the future and symptoms of depression. Many reported a scarcity of information and negative interactions with healthcare, including insensitive language and unprofessional curiosity about the child’s condition. Communication about the diagnosis by healthcare providers is a critical issue, as discussed by Johns et al. [16].

Several of the birth experiences reveal a lack of guidance and support from health staff on the basic management/care of the newborns and breastfeeding, aggravating the psychological situation of the caregivers and families. Costa et al. [30] describe the challenges that feeding an infant with special needs implicate, based on narratives from caregivers of children with craniofacial microsomia. Authors identified a lack of information and support from healthcare members as a challenge.

Faced with a lack of clear and opportune information and guidance, caregivers in this study turned into resilience strategies such as actively seeking information on their own. As reported by Johns et al. [15], caregivers of children with microsomia identified online support websites as their most important source of support. These authors highlight the significance of timely, high-quality, accessible, and differential care for children with CCAs and their families, in which understanding the psychoemotional experiences of patients and caregivers is central. For the adjustment in Caregivers of Children with Craniofacial Microsomia, Stock et al. [29] included the next as components of a framework: 1) Diagnostic experiences: pregnancy and birth, communication about the diagnosis by healthcare team, emotional responses, and information-seeking behaviors; 2) Child health and healthcare experiences: feeding, child’s physical health, burden of care, medical decision-making, surgical experiences, and quality of care; 3) Child development: cognition and behavior, educational and social experiences, emotional well-being; and 4) Family functioning: parental well-being, relationships, coping strategies, and personal growth.

Difficulties accessing specialized programs are described by caregivers in our research, when their children´s health progress depends on timely surgeries, therapies, and treatments. This situation has been also reported by Sawasdipanich et al. [31] in Thailand. As authors have seen, caregivers of children with CCAs must navigate complex, bureaucratic systems that hinder treatment. Johns et al. [15] also reported challenges in accessing intervention programs and locating experienced surgeons, as well as unmet care coordination needs in some experiences within a national sample of U.S. caregivers of children with microsomia. Our findings suggest an ambivalent, fragmented experience with access to institutional support for the care of children with CCAs, whose complex health conditions require timely referral pathways involving interdisciplinary teams and highly specialized consultation. These resources are restricted within institutions from the Colombian health system, where poor coordination in service delivery and fragmentation of care is often observed [32]. In Cali´s experiences, the limited access to specialists is more noticeable; consequently, non-governmental organizations have been an alternative for some families. Developing adjustments to care pathways and consolidating guidelines that address patients’ and their families’ differential needs are imperative, according to the findings of this study according to the findings of this study, when positive healthcare support experiences described by participants enlighten the way: services that succeeded in creating clear and organized pathways and specialists committed to the well-being of children and their families.

Although they have support resources such as their couples, their families, and their sons, almost always caregivers assume an active role in monitoring their children’s health, which involves managing administrative procedures, coordinating medical appointments, and supervising treatments. These actions are commonly aimed at achieving timely and continuous access to diagnostics, medical and therapeutic services. Their challenges may be alleviated by healthcare teams, reducing the burden of care, using a warm, family-centered supporting style, promoting coping, and linking caregivers to educational and support services [16].

As told in the previous section, in both settings, Bogota and Cali, most caring responsibilities fall almost exclusively on mothers, who not only provide direct care for their children but also carry out all or most household tasks. This double burden is closely linked to traditional gender norms that continue to position women as natural caregivers and household managers—often without support or equitable redistribution—a finding consistent with those of other authors such as Macedo et al. [33] and Johns et al. [16]. The caregiving experience of the participants in our study goes far beyond functional tasks; it is deeply intertwined with women’s identity, emotions, and life choices. For example, work-life is largely shaped by their role as caregivers, as they postpone personal goals. Caregiving also affects their physical and mental well-being, an aspect especially noted in Bogota´s accounts, possibly due to the difficulties in securing family support in a large city.

Multidisciplinary teams can play a significant role in supporting positive adjustment in patients affected by craniofacial conditions and their families from birth [16,29,30]. Patient-and-family-centered pathways for children with craniofacial alterations must be multidisciplinary, structured, and coordinated. Equally, these must be managed by a team of specialists having as a common responsibility defining key stages, phases, roles, and clinical management protocols driven to solve the differential health needs of children and their families.

## Conclusions

Delayed diagnoses limited high-quality communication and guidance, and system fragmentation, reflected in restricted access to specific adaptations and specialized care for children with CCAs, as well as the absence of adequate psychosocial support for families, emerged as common structural problems in this study when gendered caregiving burden is evident.

The healthcare system must promote an important transformation process, beginning with a comprehensive situation analysis of CCA’s health care, considering patients’ and their families’ needs. Strategies are required to enable caregivers—and subsequently, the children and adolescents themselves—to navigate structured, continuous, flexible, and timely care pathways tailored to their specific needs. Psychosocial and gender-sensitive strategies focused on alleviating the emotional burden of caregiving from diagnosis onward within a patient and family-centered care model are decisive. Improving comprehensive CCAs training for healthcare personnel and consolidating guidelines addressing patients’ and their families´ differential needs are suggested to contribute to the implementation of inclusive health programs in public-provided high-complexity care entities in both geographic settings where this research has been implemented.

The training of healthcare professionals—beginning in academia—should include specialized clinical communication and psychosocial skills for the humanized management of children with CCAs, providing person-and family-centered care that recognizes and supports the emotional, informational, and practical challenges caregivers face. This applies across all levels of complexity. Specialist and generalist healthcare professionals require adequate academic preparation and training for managing CCAs in children, particularly in terms of guidance, communication, education, and accompaniment throughout the care process, implementing gender-sensitive and patient-and-family-centered multidisciplinary approaches. It contributes to the development of inclusive health programs that address the differential needs of children with CCAs and their families, including early diagnosis.

This study captured caregivers’ challenges, revealing psychological and social burdens and their implications for healthcare delivery and family support. We hope these findings motivate future research. Exploring the lived experiences of children and adolescents with CCAs by delving deeper into their support needs in healthcare, as well as identifying successful family-centered care interventions for children with craniofacial conditions and their caregivers, could help fill a research and practice gap.

## Supporting information

S1 Appendix Contains the semi-structured interviews scripts for individual interviews and for focus group interviews. (DOCX)
