# Supplementary material for: Experiences of family caregivers regarding the health of children with congenital craniofacial anomalies in Colombia
Source: PLOS Glob Public Health. 2026 Sep 2;6(9):e0006398. doi: 10.1371/journal.pgph.0006398 (PMC13537679; doi:10.1371/journal.pgph.0006398)
Supplement: Supplementary file 1 [file pgph.0006398.s001.docx]

**S1 APPENDIX**

**Semi-structured interviews scripts**

**Semi-structured interviews scripts for individual interviews**

| **Themes** | **Questions** |
| --- | --- |
| Diagnosis and its impact on the family | **Open question: Please tell me about the family's experience since XXX's (child´s name) diagnosis.**  *Explore: What is the background of the child's birth, how the CCA was detected; What the experience of the child's birth was like; How the child was cared for at birth; Who informed the caregiver about the diagnosis and what they explained; What happened after the diagnosis was made public; How the caregiver felt and how coped with the situation; Who supported the caregiver at that time; How family members reacted, and what changes have occurred in the family since the child´s birth.*  *If the child was already born by the time the COVID-19 pandemic began, explore what the experience of caring for the child was like during the pandemic.* |
| Experiences with the healthcare team and the health system | **Open question: Please tell me about your experiences with the medical team** **that have treated XX's (child´s name) and with the health system since your child was born.**  *Explore: What the caregiver knows or what has been told about the child´s health condition and health needs, What the medical care process has been for the child's condition, and what kind of treatments the child has received; How has been the experience with the healthcare providers that have treated him, and with the health system from birth to the present. How her/his experiences were with the health system.* |
| Caregivers’ role and daily caring activities | **Open question: Please tell me about your daily life as a caregiver of a child with CCA.**  *Explore:* *What activities caregivers do at home daily; What their experience is like caring for the child; Who supports them with the child´s care; What aspects does the participant consider the most critical in her/his experience as caregiver for the child with* CCA*; How caring for the child has impacted her/his live; What activities or actions help her/his to feel better; What are her/his expectations for the future.* |

**Semi-structured interviews scripts for Focus Group Interviews**

| **Themes** | **Questions** |
| --- | --- |
| Diagnosis and its impact on the family | **Open question: Let's talk about the families’ experiences since the detection of** **CCA.**  *Explore: What was the experience like with the diagnosis; How did they cope with the situation; Who supported them at that time? What changes have occurred in the family since the birth of their children with CCA; What was the experience of caring for their children with CCA like during the pandemic.* |
| Experiences with the medical team and the health system | **Open question: Let's talk about your experiences with the medical team** **and with the health system since your children were born.**  *Explore: How and when they learned of the diagnosis; What their experiences were with their children's births and the care their children received at birth; What they know or have been told about their children's condition; How their experiences were with the* *healthcare team that have treated children from birth to the present. How their experiences were with the health system.* |
| Caregivers’ role and daily caring activities | **Open question: Let's talk about your daily activities as caregivers of a child with** **CCA.**  *Explore:* *What activities caregivers do at home daily; What their experience is like caring for the child; Who supports them with the child´s care; What aspects do they consider the most critical in their experience as caregivers for the child with* *CCA; How caring for the child has impacted their lives; What activities or actions help them feel better; What are their expectations for the future.* |
