# Supplementary material for: Experiences of family caregivers regarding the health of children with congenital craniofacial anomalies in Colombia
Source: PLOS Glob Public Health. 2026 Sep 2;6(9):e0006398. doi: 10.1371/journal.pgph.0006398 (PMC13537679; doi:10.1371/journal.pgph.0006398)
Supplement: Supplementary file 2 [file pgph.0006398.s004.docx]

**Response to** **Reviewers**

Dear Editors and Reviewers,

It´s a pleasure to greet you. Thank you for your insightful commentaries. We have carefully addressed all points and revised the manuscript accordingly. We present a table to respond to your observations.

| **Journal Requirements** | **Authors responses** |
| --- | --- |
| 1. In the online submission form, you indicated that “Data available on request.”.  All PLOS journals now require all data underlying the findings described in their manuscript to be freely available to other researchers, either  1. In a public repository,  2. Within the manuscript itself, or  3. Uploaded as supplementary information. | In accordance with PLOS journals policies, we share our data stored in a public repository: You can access supplementary material at: Lafaurie Villamil, María Mercedes; Vargas-Escobar, Lina Maria; González Carrera, María Clara; Rengifo Reina, Herney Alonso (2025), “Transcripts of focus groups and interviews. Experiences of family caregivers regarding the health of children with congenital craniofacial conditions in two cities in Colombia: a qualitative study”, Mendeley Data, V1, doi: 10.17632/63nxhypnsz.1 |
| Please ensure that your Ethics Statement is available in its entirety at the beginning of your Methods section, under a subheading 'Ethics Statement'. | Ethics Statement is available in its entirety at the beginning of the Methods section: Pag 8, line 158. |
| Please ensure that the Title in your manuscript file and the Title provided in your online submission form are the same. | For some reason, two words of the title still appear to run together. In the submission process, we tried to solve it. We will persist in resolving this. |
| We do not publish any copyright or trademark symbols that usually accompany proprietary names, eg (R), (C), or TM (e.g. next to drug or reagent names). Please remove all instances of trademark/copyright symbols throughout the text, including MAXQDA®, Google Meet®. | It has been solved. Files: 44, 54, 264, 289, 294. |
| Abstract  Line 36: Replace “primary caregivers” with “family caregivers” to better reflect the study population and maintain consistency throughout the manuscript.  Line 42: Consider replacing “semi-structured interviews and focus groups” with “individual interviews and focus group discussions,” as semi-structured interviewing is a data collection approach that may be used in both methods.  Lines 45–50: The statement that findings highlighted the “emotional complexity” of caregiving is too narrow. The findings extend beyond emotional challenges and include issues related to diagnosis, communication, healthcare access, service fragmentation, caregiving burden, and coping mechanisms. | Adjustments have been made. Lines: 36, 42, 45-50. |
| Introduction The introduction requires **substantial strengthening to better justify the study** and establish its contribution to the literature. • The introduction focuses primarily on the clinical classification and epidemiology of CCAs. **Greater emphasis should be placed on caregivers’ experiences, caregiving burden, healthcare navigation, psychosocial consequences, and quality-of-life implications.** • The literature review should include both **quantitative and qualitative studies examining caregiver experiences,** emotional burden, healthcare access, coping mechanisms, and support needs. • Additional epidemiological data are needed regarding the prevalence of CCAs, healthcare utilization, caregiver burden, and service needs. • **The** **research gap is not sufficiently articulated**. The authors should clearly explain what remains unknown regarding caregivers’ experiences, particularly within the Colombian context, and how this study addresses these gaps. • **The study objective is overly broad.** Consider presenting a general aim supported by more specific objectives that clearly identify the aspects of caregivers’ experiences being explored. | **Adjustments have been made.** Caregivers’ experiences, caregiving burden, healthcare navigation, psychosocial consequences, and quality-of-life implications are emphasized to better understand the problem. New quantitative and qualitative studies examining caregiver experiences, emotional burden, healthcare access, coping mechanisms, and support needs have been included. Lines: 99-141, 148-157.  We added epidemiological data regarding the prevalence of CCAs, including Colombian data. Lines 82-91. Caregivers’ burden and service needs have been included. Lines: 99-141. Healthcare utilization data has been difficult to find.  The arguments on the pertinence of the study and research gap have been reviewed. The study objective and aspects of caregivers’ experiences being explored in Colombian context have been clarified. Lines148-157. |
| Study **Design and Philosophical Alignment** The rationale for selecting Giorgi’s descriptive phenomenology should be strengthened. The authors should explain why this approach was chosen over other qualitative methodologies and how it aligns with the study objectives. • **A major concern is the apparent inconsistency between Giorgi’s descriptive phenomenology and thematic analysis**. The manuscript states that the study is grounded in Giorgi’s phenomenology but analyzes data using thematic analysis. These approaches are based on different analytical traditions and require clarification. • **If thematic analysis was adapted within a phenomenological framework, the authors should explicitly explain how this was accomplished and how the analytical procedures remained consistent with phenomenological principles.** • The analytical steps described do not correspond to Giorgi’s method. The authors should either revise the methodological description or justify their chosen analytical strategy more clearly. | **We understand your observations and find it relevant that there is consistency between the conceptual framework and the type of analysis implemented**. We found Giorgi´s descriptive phenomenology very interesting, especially regarding its approach to the description of lived experiences. But we saw that the intense analytical labor proposed by the author and his collaborators could make the analysis process difficult considering a sample of 25 participants. For this reason, we preferred to utilize a thematic analysis. **To guarantee coherence between method and analytical model**, we decided to align the methodology with Sundler et al.'s proposal, which not only presents a thematic analysis model but also a valuable conceptual framework on descriptive phenomenology. Lines 165-180. **About the analytical process, adjustments have been made**. Lines: 175-180, 279-287, 317-326. |
| Sampling and Participants  Although purposive sampling is mentioned, the rationale for participant selection is insufficiently described.  • **Characteristics of caregivers are largely absent**. Information such as age, education, marital status, occupation, socioeconomic status, relationship with child, and caregiving duration is important to contextualize findings and explore variation in experiences.  • The sample description currently focuses mainly on children’s characteristics. **Greater attention should be given to describing the caregivers themselves.** | Adjustments have been made. We **added information on participants' characteristics** such as age, educational levels, family type, occupation, relationship with the child, and caregiving support resources. Children ‘s gender data that doesn´t correspond to the prevalences in other settings have been discussed. Lines: 204-220 and 241-242. |
| Data Collection  The interview guide should be described in greater detail. Consider including the interview guide as an appendix.  • The **types of questions used**, probes employed, and the progression of interviews and focus groups should be outlined more clearly.  • **Saturation requires further explanation**. Describe how saturation was assessed, who determined that saturation had been reached, and what criteria were used. | Adjustments have been made.  The **interview guide has been included as an appendix** (Line 256). We expanded the information about the interview guide (lines 251-256), the recruitment period of the study (line 257) and the saturation process (lines 188-196). |
| Data Analysis and Trustworthiness  **The analysis process should be presented in a more systematic and transparent manner**, including coding procedures, development of categories, and generation of themes.  • **Consider providing a table** illustrating the analytical process from codes to categories and themes.  • The section on trustworthiness should be separated from the data analysis section.  • **Trustworthiness procedures should be described more comprehensively,** including reflexivity, audit trail, researcher positioning, triangulation, and strategies used to enhance credibility, dependability, confirmability, and transferability.  • Line 181: Reliability is reported as being ensured through sample characterization; however, **caregiver characteristics are not adequately presented.** | Adjustments have been made. As suggested, **we elaborated a table illustrating the analytical process** from categories, themes and subthemes. Line 324.  **The section on trustworthiness has been separated** from the data analysis section (lines 290-301).  **Trustworthiness procedures have been described more comprehensively**, expanding the strategies used to enhance credibility, dependability, confirmability, and transferability. Lines 291-303.    **Reflexivity as a trustworthiness practice has been included** in the Data Analysis sub-chapter and Methodological rigor (Lines 284-287, 297-298)  **Caregiver characteristics have been improved**.  **Strengths and limitations of the study have been expanded**. Lines: 305-313. |
| Results  The results section contains rich data and meaningful participant quotations. However, several structural and analytical issues should be addressed.  **Organization of Themes**  The organization of themes closely mirrors the interview guide domains, **raising concerns regarding whether themes truly emerged from participants’ experiences or were largely predetermined by the interview structure.**  • This issue should be clarified, particularly given the phenomenological orientation of the study.  • Consider **reorganizing the themes to reduce overlap and improve conceptual coherence.** For example: Theme 1: Diagnosis and Impact on Family  o Diagnostic challenges (late diagnosis, communication failures, pessimistic prognoses)  o Emotional consequences (guilt, depression, uncertainty)  o Coping and resilience (information seeking, spirituality, family support)  **• Such restructuring would provide** a more coherent narrative and better reflect the progression of caregivers’ experiences.  Depth of Analysis  Findings remain largely descriptive. Greater explanation is needed regarding the meaning of each theme and how participants experienced these situations.  • For example, the subtheme **“Failures in communication and information delivery”** discusses deficiencies in information quality, timeliness, and clarity but does not adequately explain the communication problems themselves or their consequences.  • Similar concerns apply to several other themes and subthemes.  Presentation of Findings  • **Indicate the relative frequency of findings** where appropriate (e.g., many participants, several participants, only a few participants). This helps readers appreciate the prominence of different experiences.  • **Differences between Bogotá and Cali** are insufficiently explored.  • **Variations according to caregivers’ and children’s characteristics are not examined.**  • **Line 270: Information-seeking behavior should not automatically be interpreted as a coping strategy**. It may also represent dissatisfaction with information provided by healthcare professionals or a response to unmet informational needs.  Experiences with Healthcare  • **The first four subthemes (care pathways, specialized care, therapeutic services, and COVID-19-related barriers) could be reorganized** under broader concepts such as healthcare system fragmentation, referral inefficiencies, and organizational barriers.  • This would strengthen the analytical contribution of the findings.  • The **reasons underlying restricted access to therapeutic services should be explored in greater detail.**  • The subtheme “Angels in White Coats” is not an appropriate scientific title unless it directly reflects participants’ language. Consider replacing it with a more neutral title such as “**Positive Experiences with Healthcare Professionals.”**  • **The role of non-governmental organizations should be described more thoroughly, including the** specific forms of support provided.  **Caregiver Role and Daily Care**  • **Consider consolidating findings related to emotional burden and psychological impact to reduce** redundancy across themes.  • **The role of fathers is** almost entirely absent from the analysis. Whether this reflects participant experiences, cultural norms, or caregiving patterns should be identified. Gendered caregiving burden emerges as a prominent finding and deserves stronger analytical treatment.  **• The social consequences of caregiving and living with CCAs are not sufficiently explored.**  • **Differences between regions** remain underdeveloped. | **This chapter has been reorganized according to suggestions:** reordering themes and subthemes and their denominations to reduce the overlap and improve conceptual coherence, incorporating new information, and trying to get over the descriptive emphasis in the narrative. Lines 414-670    To get information, we had conversations with caregivers to respond to the three general categories that guided the study, based on open questions, as the interview guides reveal (Appendix 1). Thus, the **themes and subthemes are emergent.**    The **relative frequency** of findings has been included.    **Although adjustments are made and Giorgi’s descriptive phenomenology is neither the conceptual framework nor the type of analysis in this manuscript version, caregivers’ experiences have been better organized, explained and evidenced.**  **“Failures in communication and information delivery**” have been discussed.    **Differences between Bogotá and Cali** have been presented. In Cali, the lack of access to specialists is noticeable, while in Bogotá, physical and mental strain emerged. Living in a big city with more difficulties in getting family support may explain this situation. Lines 532-535, 611-615, 700, 721-722,    **Variations according to caregivers’ and children’s characteristics**:  Variations according to caregivers’ have been discussed in Participants subchapter. Lines: 204-242.    **The reduced participation of cases from some anomalies doesn´t facilitate a differential interpretation of the experience**s from the perspective of the children’s conditions. This constitutes a limitation of the study that has been declared.    **Information-seeking** has been interpreted as a resilience strategy.  The **reasons underlying restricted access to therapeutic services** have been explored in greater detail.    **The subtheme “Angels in White Coats**” has been replaced as suggested.    The **role of non-governmental organizations** has been described more thoroughly,      **Caregiver Role and Daily Care**: This subchapter has been reorganized as suggested. Fathers were included; financial aspects, as a critical issue in the experience of caregivers, have been included. The social consequences of caregiving have been discussed at different points and in the discussion: Lines 542- 651, 719. |
| Discussion  **The discussion requires substantial revision and restructuring**. At present, it reads largely as a repetition of the results accompanied by supporting citations. The discussion should move beyond description and provide deeper interpretation of the findings.  • Findings are frequently **restated and linked to previous studies without adequate explanation** of why these experiences occur or what underlying mechanisms may explain them.  • **The discussion lacks a clear organizing framework** and proceeds theme by theme without developing an overarching interpretation of caregivers’ experiences.  • **Consider developing a conceptual narrative that follows caregivers’ journeys from diagnosis, emotional responses, coping strategies, interactions with healthcare systems, and long-term caregiving responsibilities.**  • A conceptual **model linking these experiences would significantly strengthen the manuscript**.  • Several important findings require deeper interpretation:  • **The emotional impact of diagnosis** and delayed diagnosis.  • **Communication failures and their** consequences.  • **Caregiver resilience and active problem-solving.**  • **Fragmentation of healthcare services and its effect on families.**  **• Gender inequality and the disproportionate burden placed on mothers**.  • Positive experiences with healthcare providers and their role in supporting adaptation.  • Given that the study claims to be grounded in Giorgi’s descriptive phenomenology, the discussion should engage more directly with the meaning of the lived experience. Questions that remain largely unanswered include:  • How did caregiving reshape caregivers’ **identities, roles, priorities, and relationships**?  • How did participants make sense of their experiences?  • What **meanings did they attribute to caregiving?**  • How did these experiences **transform their daily lives?**  • The discussion should also provide a **more critical examination of healthcare system** deficiencies in responding to the needs of children with CCAs and their families.  • Finally, the last paragraph primarily contains recommendations for healthcare professionals and educational institutions. **These recommendations are better suited to the conclusion** or a dedicated implications section rather than the discussion itself. | **Discussion**  **The discussion has been revised and restructured, according to suggestions**. A conceptual narrative that follows caregivers’ journeys from diagnosis, emotional responses, coping strategies, interactions with healthcare systems, and long-term caregiving responsibilities, has been considered.  As said, although adjustments are made and the conceptual framework has been changed, caregivers’ **experiences have been better organized, explained, and evidenced**. Lines: 642-652, 720-725.  **The conceptual framework** from Johns et al. (15) and Stock et al. (29) approaches helped us in developing a consistent narrative. Lines: 677-687.  The emotional impact of diagnosis and delayed diagnosis, Communication failures and their consequences, Caregiver resilience and active problem-solving, Fragmentation of healthcare services and its effect on families and  Gender inequality and the disproportionate burden on mothers are **topics that have been included in the discussion.** Lines: 653-673, 707-732  With the new elements that we integrated we made a **more critical examination of healthcare system**. We included positive experiences. 642-652, 668-673, 668-706, 728-732. |
| Conclusions  The conclusion generally reflects the findings but includes recommendations that are broader than the data presented.  • Recommendations should be more explicitly grounded in the study findings.  • **Consider emphasizing the key contribution of the study and the implications for healthcare delivery, caregiver support, and future research.** | Conclusions have been adjusted. Recommendations in the discussion have been replaced in this section. The key contribution of the study and the implications for healthcare delivery, caregiver support, and future research have been included. 739-741, 750-762. |
