# Supplementary material for: Experiences of family caregivers regarding the health of children with congenital craniofacial anomalies in Colombia
Source: PLOS Glob Public Health. 2026 Sep 2;6(9):e0006398. doi: 10.1371/journal.pgph.0006398 (PMC13537679; doi:10.1371/journal.pgph.0006398)
Supplement: Supplementary file 3 [file pgph.0006398.s002.doc]

PGPH-D-26-01209

Comments

This manuscript addresses an important and sensitive topic by exploring the experiences of family caregivers of children with congenital craniofacial anomalies (CCAs) in Colombia. The study covers a range of craniofacial conditions and provides valuable insights into the emotional, social, and healthcare-related challenges faced by caregivers.

The findings have the potential to inform healthcare policies, family-centered care models, and service delivery for children with CCAs and their families. The manuscript demonstrates several strengths, including the inclusion of caregivers from two geographical regions and the use of qualitative methods to explore a relatively under-researched topic. However, substantial revisions are required before the manuscript can be considered for publication.

Major concerns relate to the conceptualization of the study, methodological coherence, analytical depth, organization of findings, and interpretation of results.

**Abstract**

Line 36: Replace “primary caregivers” with “family caregivers” to better reflect the study population and maintain consistency throughout the manuscript.

Line 42: Consider replacing “semi-structured interviews and focus groups” with “individual interviews and focus group discussions,” as semi-structured interviewing is a data collection approach that may be used in both methods.

Lines 45–50: The statement that findings highlighted the “emotional complexity” of caregiving is too narrow. The findings extend beyond emotional challenges and include issues related to diagnosis, communication, healthcare access, service fragmentation, caregiving burden, and coping mechanisms.

**Introduction**

The introduction requires substantial strengthening to better justify the study and establish its contribution to the literature.

- The introduction focuses primarily on the clinical classification and epidemiology of CCAs. Greater emphasis should be placed on caregivers’ experiences, caregiving burden, healthcare navigation, psychosocial consequences, and quality-of-life implications.
- The literature review should include both quantitative and qualitative studies examining caregiver experiences, emotional burden, healthcare access, coping mechanisms, and support needs.
- Additional epidemiological data are needed regarding the prevalence of CCAs, healthcare utilization, caregiver burden, and service needs.
- The research gap is not sufficiently articulated. The authors should clearly explain what remains unknown regarding caregivers’ experiences, particularly within the Colombian context, and how this study addresses these gaps.
- The study objective is overly broad. Consider presenting a general aim supported by more specific objectives that clearly identify the aspects of caregivers’ experiences being explored.

**Methods**

Several methodological issues require clarification.

*Study Design and Philosophical Alignment*

The rationale for selecting Giorgi’s descriptive phenomenology should be strengthened. The authors should explain why this approach was chosen over other qualitative methodologies and how it aligns with the study objectives.

- A major concern is the apparent inconsistency between Giorgi’s descriptive phenomenology and thematic analysis. The manuscript states that the study is grounded in Giorgi’s phenomenology but analyzes data using thematic analysis. These approaches are based on different analytical traditions and require clarification.
- If thematic analysis was adapted within a phenomenological framework, the authors should explicitly explain how this was accomplished and how the analytical procedures remained consistent with phenomenological principles.
- The analytical steps described do not correspond to Giorgi’s method. The authors should either revise the methodological description or justify their chosen analytical strategy more clearly.

*Sampling and Participants*

Although purposive sampling is mentioned, the rationale for participant selection is insufficiently described.

- Characteristics of caregivers are largely absent. Information such as age, education, marital status, occupation, socioeconomic status, relationship to the child, and caregiving duration is important to contextualize findings and explore variation in experiences.
- The sample description currently focuses mainly on children’s characteristics. Greater attention should be given to describing the caregivers themselves.

*Data Collection*

The interview guide should be described in greater detail. Consider including the interview guide as an appendix.

- The types of questions used, probes employed, and the progression of interviews and focus groups should be outlined more clearly.
- Saturation requires further explanation. Describe how saturation was assessed, who determined that saturation had been reached, and what criteria were used.

Data Analysis and Trustworthiness

The analysis process should be presented in a more systematic and transparent manner, including coding procedures, development of categories, and generation of themes.

- Consider providing a table illustrating the analytical process from codes to categories and themes.
- The section on trustworthiness should be separated from the data analysis section.
- Trustworthiness procedures should be described more comprehensively, including reflexivity, audit trail, researcher positioning, triangulation, and strategies used to enhance credibility, dependability, confirmability, and transferability.
- Line 181: Reliability is reported as being ensured through sample characterization; however, caregiver characteristics are not adequately presented.

**Results**

The results section contains rich data and meaningful participant quotations. However, several structural and analytical issues should be addressed.

*Organization of Themes*

The organization of themes closely mirrors the interview guide domains, raising concerns regarding whether themes truly emerged from participants’ experiences or were largely predetermined by the interview structure.

- This issue should be clarified, particularly given the phenomenological orientation of the study.
- Consider reorganizing the themes to reduce overlap and improve conceptual coherence. For example: Theme 1: Diagnosis and Impact on Family
  - Diagnostic challenges (late diagnosis, communication failures, pessimistic prognoses)
  - Emotional consequences (guilt, depression, uncertainty)
  - Coping and resilience (information seeking, spirituality, family support)
- Such restructuring would provide a more coherent narrative and better reflect the progression of caregivers’ experiences.

*Depth of Analysis*

Findings remain largely descriptive. Greater explanation is needed regarding the meaning of each theme and how participants experienced these situations.

- For example, the subtheme “Failures in communication and information delivery” discusses deficiencies in information quality, timeliness, and clarity but does not adequately explain the communication problems themselves or their consequences.
- **Similar concerns apply to several other themes and subthemes**.

*Presentation of Findings*

- Indicate the relative frequency of findings where appropriate (e.g., many participants, several participants, only a few participants). This helps readers appreciate the prominence of different experiences.
- Differences between Bogotá and Cali are insufficiently explored.
- Variations according to caregivers’ and children’s characteristics are not examined.
- Line 270: Information-seeking behavior should not automatically be interpreted as a coping strategy. It may also represent dissatisfaction with information provided by healthcare professionals or a response to unmet informational needs.

Experiences with Healthcare

- The first four subthemes (care pathways, specialized care, therapeutic services, and COVID-19-related barriers) could be reorganized under broader concepts such as healthcare system fragmentation, referral inefficiencies, and organizational barriers.
- This would strengthen the analytical contribution of the findings.
- The reasons underlying restricted access to therapeutic services should be explored in greater detail.
- The subtheme “Angels in White Coats” is not an appropriate scientific title unless it directly reflects participants’ language. Consider replacing it with a more neutral title such as “Positive Experiences with Healthcare Professionals.”
- The role of non-governmental organizations should be described more thoroughly, including the specific forms of support provided.

*Caregiver Role and Daily Care*

- Consider consolidating findings related to emotional burden and psychological impact to reduce redundancy across themes.
- The role of fathers is almost entirely absent from the analysis. Whether this reflects participant experiences, cultural norms, or caregiving patterns should be identified. Gendered caregiving burden emerges as a prominent finding and deserves stronger analytical treatment.
- The social consequences of caregiving and living with CCAs are not sufficiently explored.
- Differences between regions remain underdeveloped.

**Discussion**

The discussion requires substantial revision and restructuring. At present, it reads largely as a repetition of the results accompanied by supporting citations. The discussion should move beyond description and provide deeper interpretation of the findings.

- Findings are frequently restated and linked to previous studies without adequate explanation of why these experiences occur or what underlying mechanisms may explain them.
- The discussion lacks a clear organizing framework and proceeds theme by theme without developing an overarching interpretation of caregivers’ experiences.
- Consider developing a conceptual narrative that follows caregivers’ journeys from diagnosis, emotional responses, coping strategies, interactions with healthcare systems, and long-term caregiving responsibilities.
- A conceptual model linking these experiences would significantly strengthen the manuscript.
- Several important findings require deeper interpretation:
- The emotional impact of diagnosis and delayed diagnosis.
- Communication failures and their consequences.
- Caregiver resilience and active problem-solving.
- Fragmentation of healthcare services and its effect on families.
- Gender inequality and the disproportionate burden placed on mothers.
- Positive experiences with healthcare providers and their role in supporting adaptation.
- Given that the study claims to be grounded in Giorgi’s descriptive phenomenology, the discussion should engage more directly with the meaning of the lived experience. Questions that remain largely unanswered include:
- How did caregiving reshape caregivers’ identities, roles, priorities, and relationships?
- How did participants make sense of their experiences?
- What meanings did they attribute to caregiving?
- How did these experiences transform their daily lives?
- The discussion should also provide a more critical examination of healthcare system deficiencies in responding to the needs of children with CCAs and their families.
- Finally, the last paragraph primarily contains recommendations for healthcare professionals and educational institutions. These recommendations are better suited to the conclusion or a dedicated implications section rather than the discussion itself.

**Conclusions**

The conclusion generally reflects the findings but includes recommendations that are broader than the data presented.

- Recommendations should be more explicitly grounded in the study findings.
- Consider emphasizing the key contribution of the study and the implications for healthcare delivery, caregiver support, and future research.
