# Supplementary material for: Experiences of family caregivers regarding the health of children with congenital craniofacial anomalies in Colombia
Source: PLOS Glob Public Health. 2026 Sep 2;6(9):e0006398. doi: 10.1371/journal.pgph.0006398 (PMC13537679; doi:10.1371/journal.pgph.0006398)
Supplement: Peer review history

# Peer review: Decision Letter 0

**DOI:** 10.1371/journal.pgph.0006398.r001

**Authors:** Mathilde Azar

31 May 2026

PGPH-D-26-01209

Experiences of family caregivers regarding the health of children with congenital craniofacial anomaliesin Colombia

PLOS Global Public Health

Dear Dr. Lafaurie,

Thank you for submitting your manuscript to PLOS Global Public Health. After careful consideration, we feel that it has merit but does not fully meet PLOS Global Public Health’s publication criteria as it currently stands. Therefore, we invite you to submit a revised version of the manuscript that addresses the points raised during the review process.

Please submit your revised manuscript by Jul 30 2026 11:59PM. If you will need more time than this to complete your revisions, please reply to this message or contact the journal office at globalpubhealth@plos.org. When you're ready to submit your revision, log on to https://www.editorialmanager.com/pgph/ and select the 'Submissions Needing Revision' folder to locate your manuscript file.

Please include the following items when submitting your revised manuscript:

- A letter that responds to each point raised by the editor and reviewer(s). You should upload this letter as a separate file labeled 'Response to Reviewers'.
- A marked-up copy of your manuscript that highlights changes made to the original version. You should upload this as a separate file labeled 'Revised Manuscript with Track Changes'.
- An unmarked version of your revised paper without tracked changes. You should upload this as a separate file labeled 'Manuscript'.

Guidelines for resubmitting your figure files are available below the reviewer comments at the end of this letter.

As the corresponding author, your ORCID iD is verified in the submission system and will appear in the published article. PLOS supports the use of ORCID, and we encourage all coauthors to register for an ORCID iD and use it as well. Please encourage your coauthors to verify their ORCID iD within the submission system before final acceptance, as unverified ORCID iDs will not appear in the published article. *Only*  the individual author can complete the verification step; PLOS staff *cannot*  verify ORCID iDs on behalf of authors.

We look forward to receiving your revised manuscript.

Kind regards,

Mathilde Azar, PhD

Academic Editor

PLOS Global Public Health

**Journal Requirements:**

1. In the online submission form, you indicated that “Data available on request.”.

All PLOS journals now require all data underlying the findings described in their manuscript to be freely available to other researchers, either

1. In a public repository,

2. Within the manuscript itself, or

3. Uploaded as supplementary information.

This policy applies to all data except where public deposition would breach compliance with the protocol approved by your research ethics board. If your data cannot be made publicly available for ethical or legal reasons (e.g., public availability would compromise patient privacy), please explain your reasons by return email and your exemption request will be escalated to the editor for approval. Your exemption request will be handled independently and will not hold up the peer review process, but will need to be resolved should your manuscript be accepted for publication. One of the Editorial team will then be in touch if there are any issues.

2. Please ensure that your Ethics Statement is available in its entirety at the beginning of your Methods section, under a subheading 'Ethics Statement'.

3. Please ensure that the Title in your manuscript file and the Title provided in your online submission form are the same.

4. We do not publish any copyright or trademark symbols that usually accompany proprietary names, eg (R), (C), or TM  (e.g. next to drug or reagent names). Please remove all instances of trademark/copyright symbols throughout the text, including MAXQDA®, Google Meet®.

If the reviewer comments include a recommendation to cite specific previously published works, please review and evaluate these publications to determine whether they are relevant and should be cited. There is no requirement to cite these works unless the editor has indicated otherwise.

**Additional Editor Comments (if provided):**

PGPH-D-26-01209

Comments

This manuscript addresses an important and sensitive topic by exploring the experiences of family caregivers of children with congenital craniofacial anomalies (CCAs) in Colombia. The study covers a range of craniofacial conditions and provides valuable insights into the emotional, social, and healthcare-related challenges faced by caregivers.

The findings have the potential to inform healthcare policies, family-centered care models, and service delivery for children with CCAs and their families. The manuscript demonstrates several strengths, including the inclusion of caregivers from two geographical regions and the use of qualitative methods to explore a relatively under-researched topic. However, substantial revisions are required before the manuscript can be considered for publication.

Major concerns relate to the conceptualization of the study, methodological coherence, analytical depth, organization of findings, and interpretation of results.

Abstract

Line 36: Replace “primary caregivers” with “family caregivers” to better reflect the study population and maintain consistency throughout the manuscript.

Line 42: Consider replacing “semi-structured interviews and focus groups” with “individual interviews and focus group discussions,” as semi-structured interviewing is a data collection approach that may be used in both methods.

Lines 45–50: The statement that findings highlighted the “emotional complexity” of caregiving is too narrow. The findings extend beyond emotional challenges and include issues related to diagnosis, communication, healthcare access, service fragmentation, caregiving burden, and coping mechanisms.

Introduction

The introduction requires substantial strengthening to better justify the study and establish its contribution to the literature.

• The introduction focuses primarily on the clinical classification and epidemiology of CCAs. Greater emphasis should be placed on caregivers’ experiences, caregiving burden, healthcare navigation, psychosocial consequences, and quality-of-life implications.

• The literature review should include both quantitative and qualitative studies examining caregiver experiences, emotional burden, healthcare access, coping mechanisms, and support needs.

• Additional epidemiological data are needed regarding the prevalence of CCAs, healthcare utilization, caregiver burden, and service needs.

• The research gap is not sufficiently articulated. The authors should clearly explain what remains unknown regarding caregivers’ experiences, particularly within the Colombian context, and how this study addresses these gaps.

• The study objective is overly broad. Consider presenting a general aim supported by more specific objectives that clearly identify the aspects of caregivers’ experiences being explored.

Methods

Several methodological issues require clarification.

Study Design and Philosophical Alignment

The rationale for selecting Giorgi’s descriptive phenomenology should be strengthened. The authors should explain why this approach was chosen over other qualitative methodologies and how it aligns with the study objectives.

• A major concern is the apparent inconsistency between Giorgi’s descriptive phenomenology and thematic analysis. The manuscript states that the study is grounded in Giorgi’s phenomenology but analyzes data using thematic analysis. These approaches are based on different analytical traditions and require clarification.

• If thematic analysis was adapted within a phenomenological framework, the authors should explicitly explain how this was accomplished and how the analytical procedures remained consistent with phenomenological principles.

• The analytical steps described do not correspond to Giorgi’s method. The authors should either revise the methodological description or justify their chosen analytical strategy more clearly.

Sampling and Participants

Although purposive sampling is mentioned, the rationale for participant selection is insufficiently described.

• Characteristics of caregivers are largely absent. Information such as age, education, marital status, occupation, socioeconomic status, relationship to the child, and caregiving duration is important to contextualize findings and explore variation in experiences.

• The sample description currently focuses mainly on children’s characteristics. Greater attention should be given to describing the caregivers themselves.

Data Collection

The interview guide should be described in greater detail. Consider including the interview guide as an appendix.

• The types of questions used, probes employed, and the progression of interviews and focus groups should be outlined more clearly.

• Saturation requires further explanation. Describe how saturation was assessed, who determined that saturation had been reached, and what criteria were used.

Data Analysis and Trustworthiness

The analysis process should be presented in a more systematic and transparent manner, including coding procedures, development of categories, and generation of themes.

• Consider providing a table illustrating the analytical process from codes to categories and themes.

• The section on trustworthiness should be separated from the data analysis section.

• Trustworthiness procedures should be described more comprehensively, including reflexivity, audit trail, researcher positioning, triangulation, and strategies used to enhance credibility, dependability, confirmability, and transferability.

• Line 181: Reliability is reported as being ensured through sample characterization; however, caregiver characteristics are not adequately presented.

Results

The results section contains rich data and meaningful participant quotations. However, several structural and analytical issues should be addressed.

Organization of Themes

The organization of themes closely mirrors the interview guide domains, raising concerns regarding whether themes truly emerged from participants’ experiences or were largely predetermined by the interview structure.

• This issue should be clarified, particularly given the phenomenological orientation of the study.

• Consider reorganizing the themes to reduce overlap and improve conceptual coherence. For example: Theme 1: Diagnosis and Impact on Family

o Diagnostic challenges (late diagnosis, communication failures, pessimistic prognoses)

o Emotional consequences (guilt, depression, uncertainty)

o Coping and resilience (information seeking, spirituality, family support)

• Such restructuring would provide a more coherent narrative and better reflect the progression of caregivers’ experiences.

Depth of Analysis

Findings remain largely descriptive. Greater explanation is needed regarding the meaning of each theme and how participants experienced these situations.

• For example, the subtheme “Failures in communication and information delivery” discusses deficiencies in information quality, timeliness, and clarity but does not adequately explain the communication problems themselves or their consequences.

• Similar concerns apply to several other themes and subthemes.

Presentation of Findings

• Indicate the relative frequency of findings where appropriate (e.g., many participants, several participants, only a few participants). This helps readers appreciate the prominence of different experiences.

• Differences between Bogotá and Cali are insufficiently explored.

• Variations according to caregivers’ and children’s characteristics are not examined.

• Line 270: Information-seeking behavior should not automatically be interpreted as a coping strategy. It may also represent dissatisfaction with information provided by healthcare professionals or a response to unmet informational needs.

Experiences with Healthcare

• The first four subthemes (care pathways, specialized care, therapeutic services, and COVID-19-related barriers) could be reorganized under broader concepts such as healthcare system fragmentation, referral inefficiencies, and organizational barriers.

• This would strengthen the analytical contribution of the findings.

• The reasons underlying restricted access to therapeutic services should be explored in greater detail.

• The subtheme “Angels in White Coats” is not an appropriate scientific title unless it directly reflects participants’ language. Consider replacing it with a more neutral title such as “Positive Experiences with Healthcare Professionals.”

• The role of non-governmental organizations should be described more thoroughly, including the specific forms of support provided.

Caregiver Role and Daily Care

• Consider consolidating findings related to emotional burden and psychological impact to reduce redundancy across themes.

• The role of fathers is almost entirely absent from the analysis. Whether this reflects participant experiences, cultural norms, or caregiving patterns should be identified. Gendered caregiving burden emerges as a prominent finding and deserves stronger analytical treatment.

• The social consequences of caregiving and living with CCAs are not sufficiently explored.

• Differences between regions remain underdeveloped.

Discussion

The discussion requires substantial revision and restructuring. At present, it reads largely as a repetition of the results accompanied by supporting citations. The discussion should move beyond description and provide deeper interpretation of the findings.

• Findings are frequently restated and linked to previous studies without adequate explanation of why these experiences occur or what underlying mechanisms may explain them.

• The discussion lacks a clear organizing framework and proceeds theme by theme without developing an overarching interpretation of caregivers’ experiences.

• Consider developing a conceptual narrative that follows caregivers’ journeys from diagnosis, emotional responses, coping strategies, interactions with healthcare systems, and long-term caregiving responsibilities.

• A conceptual model linking these experiences would significantly strengthen the manuscript.

• Several important findings require deeper interpretation:

• The emotional impact of diagnosis and delayed diagnosis.

• Communication failures and their consequences.

• Caregiver resilience and active problem-solving.

• Fragmentation of healthcare services and its effect on families.

• Gender inequality and the disproportionate burden placed on mothers.

• Positive experiences with healthcare providers and their role in supporting adaptation.

• Given that the study claims to be grounded in Giorgi’s descriptive phenomenology, the discussion should engage more directly with the meaning of the lived experience. Questions that remain largely unanswered include:

• How did caregiving reshape caregivers’ identities, roles, priorities, and relationships?

• How did participants make sense of their experiences?

• What meanings did they attribute to caregiving?

• How did these experiences transform their daily lives?

• The discussion should also provide a more critical examination of healthcare system deficiencies in responding to the needs of children with CCAs and their families.

• Finally, the last paragraph primarily contains recommendations for healthcare professionals and educational institutions. These recommendations are better suited to the conclusion or a dedicated implications section rather than the discussion itself.

Conclusions

The conclusion generally reflects the findings but includes recommendations that are broader than the data presented.

• Recommendations should be more explicitly grounded in the study findings.

• Consider emphasizing the key contribution of the study and the implications for healthcare delivery, caregiver support, and future research.

[Note: HTML markup is below. Please do not edit.]

Reviewers' comments:

Reviewer's Responses to Questions

**Comments to the Author**

1. Does this manuscript meet PLOS Global Public Health’s publication criteria?

Reviewer #1: Yes

Reviewer #2: Yes

**********

2. Has the statistical analysis been performed appropriately and rigorously?

Reviewer #1: N/A

Reviewer #2: Yes

**********

3. Have the authors made all data underlying the findings in their manuscript fully available (please refer to the Data Availability Statement at the start of the manuscript PDF file)?

The PLOS Data policy

Reviewer #1: No

Reviewer #2: Yes

**********

4. Is the manuscript presented in an intelligible fashion and written in standard English?

Reviewer #1: No

Reviewer #2: Yes

**********

**Reviewer #1:**  Review Comments to the Author

The topic of this manuscript does not get nearly enough attention in the literature - how families, and particularly mothers, navigate the healthcare system when caring for a child born with a craniofacial anomaly. The Colombian context makes this especially valuable, as most published work in this area comes from high-income, English-speaking settings. The two-city design is a sensible choice, and the use of both interviews and focus groups is well justified. I do think this paper has real potential, but there are a number of issues I'd ask the authors to address before it's ready.

Major Issues

The first thing I want to flag is the tension between the stated methodology and what was actually done. The authors describe the study as being grounded in Giorgi's descriptive phenomenology, but the data analysis follows the three-step thematic approach outlined by Sundler et al. (2019). These are not the same thing. Giorgi's method has specific procedural requirements, including naive reading, the identification of meaning units, and their psychological transformation, none of which are described here. I'm not saying one approach is better than the other, but the authors need to pick one and stick with it. As written, the mismatch casts doubt on the methodological foundation of the entire study.

Second, the saturation claim needs more support. The authors say they collected data until saturation was reached, but there's no explanation of how that was assessed, particularly given that interviews and focus groups were running simultaneously across two cities. More importantly, with 25 participants spread across 10 different diagnoses, it's hard to see how saturation could realistically have been achieved for the rarer conditions. This should either be better justified or acknowledged honestly as a limitation.

Third, there is no reflexivity statement anywhere in the paper. For this kind of qualitative work, that's a significant omission. The research team comes from dentistry and nursing backgrounds, and their clinical and institutional positioning will inevitably have shaped how questions were asked and how data were interpreted. Readers need that transparency. A short but honest reflexivity section would go a long way.

Fourth, the data availability statement needs work. "Data available on request" doesn't meet PLOS policy. The authors should state specifically what can be shared, who to contact, and if participant confidentiality is a barrier to full sharing, that should be spelled out explicitly rather than left implied.

Fifth, the Results section feels more like a themed collection of quotes than an analysis. The themes are clearly identified, and the participant voices are vivid, but the analytical layer is thin. For example, it's not clear how "Holding on to God's hand" and "The family as a fundamental support network" differ from each other conceptually; are these the same coping resource described differently, or genuinely distinct? The Discussion does a better job of situating the findings, but some of that interpretive work needs to happen in the Results, too.

Smaller Points

There's a typo in the abstract "provideed" on line 40, and the language needs a careful pass throughout. "Search for information as coping strategie" (line 270) and "for manage CCAs" (line 468) are two other examples. Since PLOS doesn't copyedit, this has to be clean before acceptance.

The section header "d) DISCUSSION" is capitalized differently from the others, minor but worth fixing for consistency.

Table 1 is clear and useful. One addition that would help: a note clarifying how children with more than one diagnosis were categorized.

The observation that girls outnumber boys in this sample, which runs counter to the epidemiological literature, is mentioned briefly but left unexplained. Was this a feature of the referral patterns at the participating institutions? Even a sentence of speculation would be better than leaving it hanging.

Finally, Reference 29:Chen and Kanekar (2022) appears to be an imaging paper, yet it's cited in the Discussion in the context of caregiver support and early detection. Please check that references are correctly matched to the specific claims they're supporting throughout the manuscript.

Overall

There is a solid study underneath these issues, and the findings matter for clinicians, for health system planners, and for the families themselves. The participant accounts are powerful, and the structural critique of the Colombian healthcare system that emerges from the data is well-grounded. I'd encourage the authors to revise and resubmit. My recommendation is a revision.

**Reviewer #2:**  The paper reports the experiences of caregivers while navigating through health systems and while caring for their children with congenital craniofacial anomalies in Colombia explored through qualitative study. The reporting of findings reflect the comprehensiveness in exploration, analysis and thematic presentation of findings The narratives are powerful and support the findings.

There are some minor recommendations for the authors to consider that may add value to the paper-

1) Authors have mentioned some contextual factors but if they could add a few details like whether there were any variations in ethnicity and socioeconomic conditions of caregivers and if it has shaped the healthcare seeking pathways?

2) Authors have mentioned that majority of Colombian population has been covered by General Social Security Health System (SGSSS) in the background. So if there are any findings related to how it has protected or failed to protect the caregivers from Out of Pocket Expenses(OOPE) can be added to the results section.

3) Some details in the analysis section- like the language in which interviews were conducted and in how many steps the translation and transcription to English happened?

**********

what does this mean?). If published, this will include your full peer review and any attached files.

**Do you want your identity to be public for this peer review?**  If you choose “no”, your identity will remain anonymous but your review may still be made public.

For information about this choice, including consent withdrawal, please see our Privacy Policy

Reviewer #1: **Yes:** PRADIPTA NARAYAN CHOWDHURY

Reviewer #2: No

**********

[NOTE: If reviewer comments were submitted as an attachment file, they will be attached to this email and accessible via the submission site. Please log into your account, locate the manuscript record, and check for the action link "View Attachments". If this link does not appear, there are no attachment files.]

**Figure Resubmissions:**

Attachment Submitted filename: 1. PGPH-D-26-01209_Comments.doc

# Author comment: Author response to Decision Letter 1

**DOI:** 10.1371/journal.pgph.0006398.r002

26 Jul 2026

Attachment Submitted filename: Response to Reviewers.docx

# Editor report: Decision Letter 1

**DOI:** 10.1371/journal.pgph.0006398.r003

**Authors:** Mathilde Azar

16 Aug 2026

Experiences of family caregivers regarding the health of children with congenital craniofacial anomalies  in Colombia

PGPH-D-26-01209R1

Dear Professor Lafaurie,

We are pleased to inform you that your manuscript 'Experiences of family caregivers regarding the health of children with congenital craniofacial anomalies  in Colombia' has been provisionally accepted for publication in PLOS Global Public Health.

Before your manuscript can be formally accepted you will need to complete some formatting changes, which you will receive in a follow up email. A member of our team will be in touch with a set of requests.

Please note that your manuscript will not be scheduled for publication until you have made the required changes, so a swift response is appreciated.

IMPORTANT: The editorial review process is now complete. PLOS will only permit corrections to spelling, formatting or significant scientific errors from this point onwards. Requests for major changes, or any which affect the scientific understanding of your work, will cause delays to the publication date of your manuscript.

If your institution or institutions have a press office, please notify them about your upcoming paper to help maximize its impact. If they'll be preparing press materials, please inform our press team as soon as possible -- no later than 48 hours after receiving the formal acceptance. Your manuscript will remain under strict press embargo until 2 pm Eastern Time on the date of publication. For more information, please contact globalpubhealth@plos.org.

Thank you again for supporting Open Access publishing; we are looking forward to publishing your work in PLOS Global Public Health.

Best regards,

Mathilde Azar, PhD

Academic Editor

PLOS Global Public Health

***********************************************************

No additional comments.

Reviewer Comments (if any, and for reference):
